# The Code Silver Exercise: a low-cost simulation alternative to prepare hospitals for an active shooter event

**DOI:** 10.1186/s41077-021-00190-0

**Published:** 2021-10-21

**Authors:** Julie J. Kim, Daniel Howes, Chantal Forristal, Andrew Willmore

**Affiliations:** 1 Department of Medicine, Division of Emergency Medicine, Lawson Research Institute, Western University, London, Ontario Canada; 2 Department of Emergency Medicine, London Health Sciences Centre, Victoria Hospital Campus, 800 Commissioners Road East, Room E1-125, London, Ontario N6A 5W9 Canada; 3 Department of Critical Care Medicine, Queens University, 76 Stuart Street, Kingston, Ontario K7L 2V7 Canada; 4 Department of Emergency Medicine, University of Ottawa, 1053 Carling Avenue, Ottawa, Ontario K1Y 4E9 Canada; 5 Ottawa Hospital Research Institute, The Ottawa Hospital, Ottawa, Ontario Canada

**Keywords:** Person with a weapon, Virtual simulation, Emergency preparedness, Training

## Abstract

Mass-shooting incidents have been increasing in recent years and Code Silver—the hospital response to a person with a weapon such as an active shooter in many Provinces or States in North America—is quickly shifting from a theoretical safety measure to a realistic scenario for which hospitals must prepare their staff. A Code Silver Exercise (CSE) involving an independent mental practice exercise with written responses to scenarios and questions, followed by a facilitated debrief with all participants, was conceptualized and trialled for feasibility and efficacy. The CSE was piloted as a quality improvement and emergency preparedness initiative in three different settings including in situ within a hospital Emergency Department or Intensive Care Unit, offsite in a large conference room workshop, and online via virtual platform. These sessions took place in 4 different cities in Canada and included 3 academic teaching hospitals. Participants of the in situ and virtual CSE completed pre- and post-simulation surveys which showed improved understanding of Code Silver protocols following participation.

The CSE is a reproducible simulation alternative, designed to operationalize a Code Silver policy at a large healthcare institution in a sustainable way. This training model can be administered in multiple settings in-person (in situ or offsite), and virtually, making it versatile and easily accessible for participants. This exercise enables participants to mentally rehearse practical responses to an active shooter in their unique work environments and to discuss ethical and medical-legal implications of their responses during a facilitated debrief with fellow healthcare providers. Implementation of a CSE for training in hospitals may help staff to create a mental schema prior to an active shooter event, and thus indirectly improve the chances of survivability in the event of a real active shooter situation.

**Supplementary Information:**

The online version contains supplementary material available at 10.1186/s41077-021-00190-0.

## Background

Mass shooting events are becoming more common in North America, and while there is still a paucity of literature into gun violence in hospitals, a study between 2000 and 2011 showed 154 hospital-based shootings in the USA [1]. This study also identified the emergency department (ED) and hospital parking lots as the most vulnerable locations for these incidents [1]. While shootings in Canadian hospitals may be less frequent, they are not an exception. Table 1 outlines Canadian hospital-related shootings and lockdowns between 2000 and 2020 and displays the sobering trend since 2014 that hospital-related shooting incidents in Canada are becoming more frequent.

**Table 1:** Canadian hospital shooting-related lockdowns since 2000–2020

Date	City	Hospital	Location (number injured or killed)
May 20, 2003	Mission, BC	Mission Memorial Hospital [2]	Ward (2)
Aug 29, 2006	Penticton, BC	Penticton Regional Hospital [3]	Ward (2)
May 7, 2007	Ottawa, ON	Children’s Hospital of Eastern Ontario and ROTEL building [4]	ED entrance and ROTEL (0)
Apr 1, 2011	Belleville, ON	Belleville General Hospital [5]	Parking lot (1)
May 14, 2014	Ottawa, ON	The Ottawa Hospital, Civic Campus [6]	Hospital washroom (1)
Jun 2, 2014	Etobicoke, ON	Etobicoke General Hospital Parking lot [7]	Parking lot (1)
May 20, 2015	Guelph, ON	Guelph General Hospital [8]	ED wait room (1)
Nov 26, 2015	Calgary, AB	Foothills Hospital [9]	ED (1)
Jun 24, 2016	North York, ON	North York General Hospital [10]	Hospital Entrance (0)
Jan 12, 2017	Grand Forks, BC	Boundary District Hospital [11]	ED (1)
Jan 17, 2017	Calgary, AB	Foothills Hospital [12]	ED entrance (1)
Oct 27, 2017	Cobourg, ON	Northumberland Hills Hospital [13]	ED (2)
May 18, 2018	Fort Erie, ON	Douglas Memorial Hospital [14]	Urgent Care (2)
Nov 19, 2018	Kingston, ON	Kingston General Hospital [15]	ED (1)

List of shooting-related hospital lockdowns as found on public news listings and media outlets [2–15]. The above list does not include hospital lockdown events related to unsubstantiated threats for an active shooter

The rise in gun-related violence and mass casualty shooting events has necessitated the development of preparedness strategies. The 2013 Hartford Consensus—created following the Sandy Hook Elementary School shooting in the USA—is a set of recommendations from a committee of experts, namely the American College of Surgeons, Federal United States Government, National Security Counsel, United States Military, Federal Bureau of Investigations, and emergency medical response organizations, to improve survivability from an active shooter event [16–19]. These recommendations include training videos and exercises supported by the US Department of Homeland Security that advocate the ‘Run, Hide, Fight’ strategy. This strategy trains individuals to react to an active shooting by leaving the area if possible, hiding if exit is not possible, and lastly, fighting if confronted by a person with a weapon. This work has more recently been adapted into recommendations for how health care facilities can incorporate active shooter incident planning into their operations plans [20].

Many hospitals around the world use a standardized system of colour codes to inform staff of emergency situations in the hospital and to activate the appropriate emergency preparedness protocols. Examples include Code Red for a fire or Code Orange for an external disaster [21]. In 2016, the Ontario Hospital Association recommended the addition of Code Silver to standardize the language, application, and approach to managing a person with a weapon such as an active shooter [21]. Previously, this response fell under a Code Purple (hostage situation). Given that hostage-taking and active shooter scenarios require very different hospital and police responses, the division into two separate codes was deemed necessary.

In an active shooter event, immediate action from front-line personnel can directly influence patient and staff survival [22]. Healthcare providers must consider their own well-being, as well as that of their patients in an emergency scenario, which means that the ‘Run, Hide, Fight’ recommendation could evoke a spectrum of personal responses depending on the individual. After the implementation of Code Silver in Ontario, many hospitals chose to recommend the strategy of ‘Run, Hide, Survive’, so as to not encourage staff to fight an assailant. Though, if one is unable to safely evacuate or hide, the aim is to survive by any means possible if facing imminent threat from a person with a weapon. Interestingly, a survey conducted in the USA revealed that more than half of the public sector would expect doctors and nurses to put themselves at risk to protect a patient, similar to the duty of a police officer or firefighter [23]. However, this is not something healthcare providers may be comfortable doing, nor is it necessarily their duty.

In March 2017, we contacted representatives from the Canadian Medical Protective Association via written and verbal communication regarding physician responsibilities in a Code Silver including potential liability issues. These communications indicated that a physician’s duty to patient care does not always take priority over the physician’s own health and safety. A physician treating a critically ill patient when a hospital Code Silver is initiated will want to use professional judgement and information available to determine whether it would be reasonable to leave a patient unattended. If a physician were later to be sued for a breach in duty to care, they would be held to the standard of a ‘reasonable physician’ in similar circumstances.

The ethical and legal ambiguity and onus on personal judgement in the event of a Code Silver makes it difficult for healthcare providers to know what is expected of them. This can compromise their ability to respond decisively in order protect themselves, their colleagues, and their patients in an active shooter situation.

## Purpose and rationale

Active shooter situations have the potential for devastating morbidity and mortality, and hospitals have a duty to train staff at their institutions for emergency preparedness procedures. Code Silver was implemented at hospitals across Ontario in 2016, yet it is unclear if hospital workers have been trained to know what actions they must take as individuals in this situation, or what is expected of them in terms of protecting their patients both ethically and medical-legally. Electronic learning modules and videos have been the easiest modalities to facilitate employee safety training in large institutions, but emergency preparedness training is not standardized for hospitals. Both computer and lecture-based techniques have been used for disaster training of health care workers, but it is difficult to determine which training interventions are effective at improving knowledge and skills to respond to a disaster [24]. Furthermore, these models seldom address the specific environments in which employees work, nor do they adequately discuss ethical, moral, and legal obligations to patient care. The way in which hospital staff react to a crisis situation is highly variable and elevated stress levels can negatively impact performance and decision making abilities [25].

Simulation is increasingly being used in healthcare to prepare trainees for emergency situations that are infrequently encountered, but where complex skills and assessments are required under dynamic conditions and stressors. Ideally, Code Silver training could be conducted via in situ simulation with simulated participants acting as shooters, patients, and hospital visitors [26–29]. However, this type of robust simulation is expensive, difficult to coordinate, and can only include a limited number of participants with each session. Furthermore, conducting a large in situ simulation of this violent nature can elicit feelings of trauma and distress to participants and can be disruptive to patient care and department flow.

Mental practice, visualization, or the use of imagery is a skill that allows one to walk through the steps of a procedure or scenario before it is encountered in real life. Mental practice has been defined as the ‘rehearsal of a skill in the absence of any physical movement’ [30]. This practice has been successfully described in the training and performance of musicians and athletes [30, 31]. More recently, mental practice in medicine was successfully implemented to improve performance of simulated medical and surgical techniques such as laparoscopic cholecystectomies, as well as during Advanced Trauma Life Support resuscitation training [32, 33]. Specific evidence-based elements of motor imagery practice were described by Collins and Holmes in 2001 with the PETTLEP model [34]. This model suggests that seven factors should be considered when designing imagery practice: Physical, Environment, Timing, Learning, Emotions, and Perspective of the person.

With these factors in mind, we developed the active shooter Code Silver Exercise (CSE). The CSE is a practical and sustainable training tool that can be administered in a variety of settings to prepare hospital staff for rare emergencies such as an active shooter situation.

## Description of the innovation

The objectives of the CSE were to (1) review the steps to activate and respond to a Code Silver in the hospital, (2) have participants mentally practise various scenarios presented within an active shooter scenario, and (3) debrief and discuss logistical and ethical considerations with colleagues in a facilitated debrief by content experts.

The CSE was piloted in three different settings: (1) an in situ exercise conducted in two EDs and one intensive care unit (ICU), (2) an exercise offsite at a conference workshop, and (3) an online virtual platform, with a virtual debrief within 1 week.

Participants were informed in advance that the CSE could involve discussion of potentially violent, emotional, or uncomfortable content matter involving active shooter scenarios, and were given the option to decline participation. Research ethics board (REB) exemptions were obtained at Kingston General Hospital, The Ottawa Hospital, and at London Health Sciences Centre (LHSC), Canada, as the CSE was administered as a quality improvement initiative. An REB exemption was not completed for the workshop CSE in Montreal, Canada, given that no data were collected from participants.

### In situ CSE

The in situ CSE was piloted in January 2017 in the Kingston General Hospital ED and ICU with senior medical residents as participants. It was repeated in February 2017 with medical residents and emergency management administrators in the ED of The Ottawa Hospital, Civic Campus.

The in situ CSE involved three parts. Part 1 was a Code Silver teaching module comprised of an approximately 20-min didactic PowerPoint presentation summarizing the background and need for a Code Silver response. It included information such as how to activate Code Silver, the ‘Run, Hide, Survive’ principles, the expected tactical police response to a Code Silver, how a Code Silver impacts other concurrent emergency codes in the hospital, and lockdown procedures. All content included in the Code Silver module was approved by emergency management leadership from respective hospital sites.

Part 2 was the in situ mental practice exercise. Each participant was given a paper booklet describing a clinical scenario involving an active shooter (see Supplemental Document). Participants were instructed to physically go to 1 of 4 specified stations in the ED or ICU and asked to write answers to 2–3 brief questions during a period of 3 min at each station. The questions focused on ‘Run, Hide, Survive’ principles, and ethical dilemmas pertaining to patient care duties versus personal safety. Questions were designed based on iterative feedback from emergency management experts and leadership personnel at Kingston General Hospital and The Ottawa Hospital. Stations were timed to mimic the pressure of a real-life scenario. Participants answered questions silently and independently, then moved to the next location.

Part 3 of the CSE was a 20-min facilitated debrief discussion during which participant answers were discussed as a group. The principles of ‘Run, Hide, Survive’ were explored in the context of participants’ specific work environment, as well as with respect to ethical and medical-legal patient care considerations. Debrief facilitators included a ED physician with fellowship experience in simulation, and/or pre-hospital emergency and disaster medicine. Local police services in Ottawa, Kingston, and London, Ontario, were also interviewed prior to the debriefs such that the facilitators were aware of what would be involved in the tactical police response to an active shooter in these respective hospitals. Correspondence from The Canadian Medical Protective Association guided any discussion of physician medical-legal liability for patient care during an extenuating circumstance such as a Code Silver. Debrief participants engaged in facilitated discussions based on case objectives following the PEARLS debriefing framework [35].

### Workshop CSE

The CSE was modified for a conference room setting and administered during a workshop at the Emergency Preparedness In Health Care Conference in Montreal, Canada in December 2018. This workshop involved physicians, nurses, and administrators from hospitals across Canada and internationally. This conference room CSE involved the same three components as the in situ CSE with some modifications. Part 1 reviewed the concept of Code Silver, but given the diverse group, did not provide specific details regarding local hospital or regional police responses. For Part 2, participants rotated through 4 stations every 3 min. Photographs of clinical areas inside an ED were posted on the wall at each station, and participants were asked to picture themselves in similar locations in their own working environments and to complete the written responses independently (see Supplemental Document)*.* This was followed by Part 3, a 20-min facilitated debrief with all participants.

### Virtual CSE

With the COVID-19 pandemic in 2020, the CSE was further adapted as a Virtual Interactive Case for ED physicians at LHSC and piloted in August 2020 [36]. Given that ED physicians at LHSC are required to complete annual electronic learning modules for emergency response procedures that include Code Silver with content usually provided in our didactic training module, we elected to remove Part 1 from the virtual case. Of note, when sessions were provided to participants with unknown or varied exposure to Code Silver training (such as the in-situ and workshop participants), the Part 1 didactic session was required to ensure all participants had baseline knowledge of how to initiate a Code Silver and the ‘Run, Hide, Survive’ steps. In the virtual CSE the ‘Run, Hide, Survive’ steps were reinforced during Part 3, the facilitated debrief. ED physicians at LHSC were invited by e-mail to participate in the interactive virtual case. Participation was voluntary, and the CSE was completed independently at a time convenient for the physician over a 1-week period on an online survey programme (SurveyMonkey) distributed via e-mail link. Each ‘page’ of the survey was equivalent to a ‘station’ in the in-person exercises. Photographs of the LHSC ED were provided on each page of the survey to orient participants as they progressed between stations and questions. Participants were encouraged to limit their time to 3 min per page, for a total completion time of 12 min, in keeping with the 3 min allotted per station in the in-person exercises. A 1-h facilitated debrief was conducted 1 week later via Zoom videoconference with voluntary participation for those who completed the online CSE.

## Results

Total participation included *n*=9 for the in-situ CSE, *n*=24 for the workshop CSE, and *n*=25 for the virtual CSE. For the voluntary debrief portion of the virtual CSE, *n*=11 physicians participated in the facilitated debrief 1 week later via Zoom videoconference.

### Survey results

**Fig. 1:**
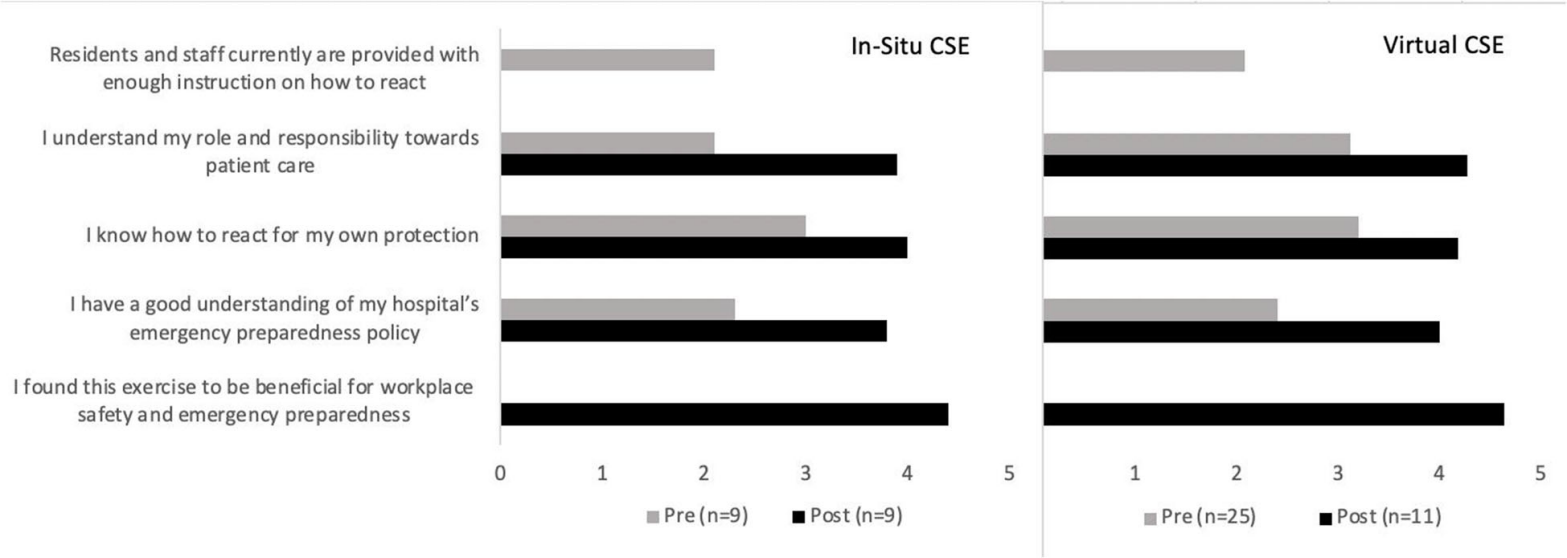
Pre- and post-survey results for in situ and virtual CSEs. Mean ratings on a Likert scale of 1 ‘strongly disagree’ to 5 ‘strongly agree’ for the above statements pertaining to situations when there is an armed and potentially dangerous person in the hospital

Figure 1 shows results from pre- and post-CSE surveys that were distributed to participants for both the in situ and virtual CSE to obtain feedback. Response rates were 100% for both surveys. A total of 34 participants completed the pre-CSE survey (in situ CSE, *n*=9; virtual CSE, *n*=25). A total of 20 participants completed the post-CSE survey (in situ CSE, *n*=9; virtual CSE, *n*=11). Note that the 14 participants in virtual CSE who did not participate in the voluntary debrief were not distributed a post-CSE survey since they had not completed both parts of the exercise, hence the difference in pre- and post-CSE survey participant numbers.

The pre-CSE survey responses indicated that only 6% (*n*=2/34) of respondents agreed (scored 4 or 5 on a 5-point Likert scale) that residents and staff are currently provided with enough instruction on how to react in a Code Silver situation. When asked if they understood their role and responsibility to patient care pertaining to Code Silver, 35% (*n*=12/34) of participants agreed pre-CSE, which improved to 80% (*n*=16/20) post-CSE. Prior to participation, 41% (*n*=14/34) agreed they would know how to react for their own protection during a Code Silver, which improved to 100% (*n*=20/20) post-CSE. When asked if participants had a good understanding of their hospital’s emergency preparedness policies for Code Silver, 18% of participants (*n*=6/34) agreed pre-CSE, while 80% (*n*=16/20) agreed post-CSE. Lastly, 100% (*n*=20/20) of participants agreed that this exercise is beneficial for workplace safety and emergency preparedness.

### Themes and lessons learned from the debrief

The debrief discussions during all CSE highlighted that there is much uncertainty and personal variability in the application of a Code Silver protocol. The dominant theme identified was that one’s instincts in an active shooter situation are highly variable from one person to the next. This held true for moral and ethical decisions as well as physical reactions and interpretation of ‘Run, Hide, Survive’. Participants agreed that the most important considerations included personal location relative to the shooter, clinical or non-clinical responsibilities, ability to secure an area (lock or barricade), and the medical acuity of their patients relative to the Code Silver threat.

Many participants found themselves questioning their medical-legal obligations during a Code Silver situation. The fact that medical-legal responsibility is very situation-specific and subjective based on what a ‘reasonable physician’ would do, further emphasizes the importance of healthcare providers having these debrief discussions prior to a Code Silver event. The CSE allows participants to gauge where their personal response fits on a spectrum compared to their colleagues, who could serve as examples of ‘reasonable physicians’ in the event of a legal case. If a participant’s responses to the CSE were not aligned with their colleagues’, they could adapt their personal Code Silver approach to be more in-line with hospital, and medical-legal expectations.

A common theme was that participants noted a new awareness of their environmental surroundings, or previous lack thereof. Interestingly, this was consistent amongst participants in all three groups including in-situ, offsite and virtual CSE. Some participants had never noticed a certain exit, stairwell, or closet within their unit. Other participants noted they never paid attention to the types of doors, locks, or considered how they might secure their unit prior to the exercise. Others identified the challenges with working in a unit that had no doors or rooms, only curtained areas. Discussion revealed that locked units may become unlocked in the event of a fire alarm being pulled, or that other concurrent codes such as a Code Blue (cardiac arrest) would then have to be put on hold until the Code Silver is cleared. Participants shared how they may barricade themselves in their areas and which tools might be the most accessible for this. These, amongst other important safety and logistical issues, were raised during debrief discussions.

## Discussion

The CSE was found to be a versatile and effective training tool. Though it was conducted in three drastically different physical locations (in situ, offsite workshop, and virtual), the overall themes elicited in the debrief sessions were very similar in all settings. The virtual CSE allowed for increased flexibility for participation. The simplicity and flexibility of the tool by utilization of mental practice makes it sustainable for larger institutions to implement without significant budget allocation or affecting patient care in clinical areas.

Many hospitals in Canada undergo an accreditation process; however, there are no standardized criteria for emergency preparedness training of hospital staff on a provincial or national level. While hospitals are faced with the responsibility to establish consistent and effective emergency preparedness strategies, the rarity of an active shooter event poses the risk that education, protocols, and policies in place are unlikely to be tested unless a real event occurs. Full-scale in situ simulation exercises have shown that identifying and reviewing latent safety threats are critical to emergency preparedness for an active shooter in the ED [26, 27]. Despite awareness of the ‘Run, Hide, Fight’ strategy, previous studies have suggested that healthcare personnel may have a more ‘patient-centric’ rather than self-preservation strategy in a Code Silver situation, and these moral and ethical considerations must be discussed prior to a real event [29, 37]. Similar to our findings, previous active shooter simulations in the ED have increased healthcare provider knowledge and confidence in feeling prepared for a Code Silver event [29].

The goal of the CSE is not to masquerade as a traditional simulation, nor is it to replace electronic learning modules or educational videos. Rather, it is to expand upon the existing active shooter training in place to improve healthcare provider preparedness and increase survivability in the event of a Code Silver situation. We recognize that our pilot sessions were done with a small number of participants; however, the CSE exercises presented in this paper demonstrate the versatile nature in the in situ, offsite, and virtual nature of delivery based on an institution’s needs. This model can be implemented for hospital-wide training and can further be designed operationally as a ‘Train the Trainer’ session such that unit managers or leaders within specific hospital departments can be trained to later facilitate ongoing CSE trainings and debrief at a departmental or unit level. Alternatively, hospital emergency management staff could run dedicated sessions tailored to each department in the hospital.

Given that a significant benefit of the CSE was in the debrief discussions of ethical, moral, and legal responsibilities for patient care, we believe physicians and nurses who are most responsible for patient care would benefit most from this additional training. However, training for all ED personnel should be considered high priority given that this is a high-risk department in the hospital [1]. The CSE was designed for smaller groups to be able to facilitate intimate discussion, ideally with a maximum of 20–30 participants. Depending on the healthcare provider background, hospital resources, and scheduling availability, a CSE can be conducted in an in situ, offsite, or virtual setting, and can be completed in under 1 h. Importantly, we believe the CSE can be used and adapted by many patient care centres to make healthcare institutions safer for patients and staff.

### Limitations

Although this CSE exercise shows promise, the scope of this pilot was unable to show lasting impact and long-term retention of knowledge using CSE. Furthermore, we cannot definitively demonstrate superiority over computer-based learning modules. However, pilot studies in medical education comparing immersive learning techniques such as simulation compared to didactic or pre-recorded computer teaching have highlighted either no difference or greater knowledge retention and engagement with immersive learning [38, 39].

The addition of time pressure with limiting each station to 3 min is not a typical feature of mental practice, which some may argue limits the effectiveness of the mental practice. However, the incorporation of realistic time pressures may have heightened participant emotion, and timing and emotion are both critical elements supported by the PETTLEP model [34]. With these elements in mind, another limitation of the exercise was that we could not specifically instruct participants how to answer the questions during the brief stations. Unlike a deliberate practice simulation for a medical procedure with a correct sequence of steps, responding to a life-threatening situation while providing patients with medical care is a complex scenario that will evoke different responses and emotions from individual participants. While participants could not be guided in their mental practice during the CSE itself, these elements were discussed during the debrief. Additionally, following the debrief discussion, we encouraged participants to synthesize their thoughts without any time pressures and mentally rehearse their individual plan should they encounter an active shooter situation in their usual work environments. This is supported by sports psychology where slower time mental rehearsal can make a powerful contribution to even very fast-action or unconscious tasks [40].

Lastly, the fidelity of a mental practice exercise may seem lower when compared to traditional physical simulation exercises. While we cannot prove that mental practice is superior to physical practice, the literature supports that the addition of mental practice can improve physical skills compared to physical practice alone [41]. Experiments in sports psychology also support that the PETTLEP model of imagery is more effective than traditional imagery models [42]. While the workshop and virtual CSE were limited in the physical and environmental components of PETTLEP, the in situ CSE incorporates all seven of these important factors. We did attempt to address this limitation by posting pictures of clinical environments on the walls at stations in the workshop CSE or in the virtual CSE. A full-scale simulation which includes actors, moulage, and firearms brings other notable challenges including significant cost, coordination with local police and hospital personnel, disruption of patient care, and clinical space. Furthermore, the protection of psychological safety for all involved must always be considered with dramatization and simulation using weapons and acted violence. Such an endeavour presents a huge logistical and financial undertaking that is not sustainable, nor does it reach the majority healthcare workers in a large institution. Future work may consider feasibility or cost-effectiveness comparisons of our innovation compared to more traditional methods of active shooter training.

## Conclusion

The CSE is an alternative to traditional simulation for an active shooter event that can be implemented in a sustainable way without affecting patient care or daily operations at large healthcare institutions. The concept of mental practice has been applied to our CSE, to impart an institutional policy in an engaging way. Where an electronic learning module or video simply delivers information to a broad audience, the CSE requires one to mentally practise and develop a construct for how to act and react in an active shooter situation. It allows staff to reflect, plan an escape route, locate safe hiding places, and consider tools for self-defence. Importantly, it educates staff in advance of a Code Silver to consider and weigh the ethical dilemma of self-protection versus patient care during an emergency. An active shooter event is an example where the physical, psychological, and legal consequences for the split-second decisions of healthcare providers require individual reflection and forethought prior to a real event.

## Supplementary Information

**Additional file 1.** Supplementary Documentation.

## Data Availability

The datasets during and/or analysed during the current study is available from the corresponding author upon reasonable request.

## Abbreviations

CSE: Code Silver Exercise
ED: Emergency department
ICU: Intensive care unit
REB: Research ethics board
LHSC: London Health Sciences Centre
