# Supplementary material for: The Code Silver Exercise: a low-cost simulation alternative to prepare hospitals for an active shooter event
Source: Adv Simul (Lond). 2021 Oct 21;6:37. doi: 10.1186/s41077-021-00190-0 (PMC8529569; doi:10.1186/s41077-021-00190-0)
Supplement: Supplementary file 1 — Additional file 1. Supplementary Documentation. [file 41077_2021_190_MOESM1_ESM.docx]

**SUPPLEMENTAL DOCUMENT**

**Code Silver Exercise**

**Participant Instructions**

- This is an independent and silent exercise.
- Each station is 3 minutes long.
- There is 1 page of questions per station, do not flip ahead.
- Answer in point form. Write down any notes, comments thoughts or questions you may have.
- When time is up, please move to your next station, flip the page and answer the questions on that page.
- If you start at Station A, your next station is B🡪C🡪D.
- If you start at Station D, your next station is A🡪B🡪C.
- When the exercise in completed, please return to the debrief room.

**YOUR GROUP: A B C D**

**START AT STATION: A B C D**

**START ON PAGE: 2 3 4 5**

**DO NOT OPEN THIS PACKAGE UNTIL THE EXERCISE HAS STARTED.**

**PLEASE DO NOT FLIP AHEAD BETWEEN STATIONS.Station A:**

You are an **Emergency Physician** ***(or insert other role****)* working in the Emergency Department on a weekday afternoon. Overhead you hear the following “Code Silver, Cafeteria. Initiate Lockdown.”

Your patient and their family member at the bedside hear this and become anxious, asking you what this means. Your patient develops severe chest pain and shortness of breath.

1. Where is the closest exit and what route would you use to exit the hospital?
2. Would you leave the hospital if the Code Silver was not in your immediate area?
3. When is it appropriate for you to leave the building when caring for sick patients?

**Station B:**

You are a **Hospital Administrator *(or insert other role****)* working in the Emergency Department on a weekday afternoon. Overhead you hear the following “Code Silver, Cafeteria. Initiate Lockdown.”

1. Describe how you would lock down your unit.
2. How would you barricade the doors to your unit or to a patient room to prevent an armed person from entering?

**Station C:**

You are a **Nurse** ***(or insert other role****)* working in the Emergency Department on a weekday afternoon. Overhead you hear the following “Code Silver, Cafeteria. Initiate Lockdown.”

1. If the unit is locked and you are instructed to hide in place, where and how would you hide from where you are standing?
2. If the fire alarm is pulled and your unit becomes unlocked, what tools would you use to secure your unit?

**Station D:**

You are a **Resident Physician** ***(or insert other role****)* working in the Emergency Department on a weekday afternoon. Overhead you hear the following “Code Silver, Cafeteria. Initiate Lockdown”

1. There is a person with a weapon (gun) threatening you or people around you. What would you use in your immediate vicinity as a potential tool or weapon for survival?
2. What is the best way to communicate that an armed person is now in your unit as opposed to the location originally announced overhead?
